# The role of vaginal palpation in motor learning of the pelvic floor muscles for women with stress urinary incontinence: study protocol for a randomized controlled trial

**DOI:** 10.1186/s13063-020-04624-4

**Published:** 2020-07-31

**Authors:** Letícia de Azevedo Ferreira, Fátima Faní Fitz, Márcia Maria Gimenez, Mayanni Magda Pereira Matias, Maria Augusta Tezelli Bortolini, Rodrigo Aquino Castro

**Affiliations:** Department of Gynaecology, Federal University of Sao Paulo (UNIFESP), Rua Napoleão de Barros, 608 – Vila Clementino, São Paulo, SP CEP 04024-002 Brazil

**Keywords:** Physical therapy modalities, Pelvic floor, Knowledge, Urinary incontinence, Randomized controlled trial

## Abstract

**Background:**

Approximately 30 to 50% of women are unable to correctly perform pelvic floor muscle (PFM) contractions. For women to benefit from a pelvic floor muscle training (PFMT) programme for stress urinary incontinence (SUI), the awareness phase of PFMT cannot be omitted. The purpose of this study is to evaluate whether vaginal palpation together with verbal instructions about PFMs and body awareness techniques helps women with SUI learn how to correctly contract the PFMs and improve their functions.

**Methods:**

This single-centre, double-blind randomized controlled trial with two intervention groups was designed following the standard protocol items for randomized interventional trials (SPIRIT). The results will be reported in a manner consistent with the Consolidated Standards of Reporting Trials (CONSORT) guidelines. Patients with SUI (*n* = 172) will be recruited. The experimental group will receive verbal instructions about PFM function and body awareness techniques together with vaginal palpation; the control group will receive similar protocol without vaginal palpation. The primary outcome includes the number of fast-twitch muscle fibres assessed by vaginal palpation and visual observation. Secondary outcomes include power and muscular endurance that will be assessed by visual observation and vaginal palpation (Oxford scale), the use of accessory muscles during the voluntary contraction of PFMs, and the self-efficacy and the expectations for the results using the self-efficacy scale of pelvic floor exercises.

**Discussion:**

This study will determine whether vaginal palpation can help women with SUI to correctly perform PFM contractions and improve their functions.

**Trial registration:**

ClinicalTrials.gov NCT 03325543. Registered on 30 November 2017. Study protocol version 1; 30 November 2020. Prospectively registered.

## Introduction

Stress urinary incontinence (SUI) is defined as an involuntary loss of urine during effortful manoeuvres, such as coughing or sneezing [1]. Pelvic floor muscle training (PFMT) is a conservative and first-line treatment for women with SUI [2] that can be included in both outpatient and home care regimens. The success rate of the outpatient regimen ranges from 60 to 75% [3, 4], and that of the home regimen ranges from 9 to 17% [3, 5]. However, in practice, approximately 30 to 50% of women are unable to correctly perform perineal muscle contractions. Some factors may contribute to this finding, such as the location of the pelvic floor muscles (PFMs), their small size, and the general lack of knowledge on the pelvic and perineal regions and their functions. In addition, women usually initially contract their gluteal, hip adductor, or abdominal muscles rather than their levator anus muscle when asked to contract their PFMs [6–9]. For women to benefit from a PFMT programme for the treatment of SUI, the awareness phase of PFMT cannot be omitted; all relevant previous studies have suggested that pelvic floor exercises improve the recruitment capacity of the musculature and its tone and individuals’ reflex coordination during effortful activities [7, 10].

The overall objective of this study is to evaluate whether vaginal palpation together with verbal instructions about the anatomy and function of the PFMs and the use of body awareness techniques helps women with SUI learn how to correctly contract the PFMs and improve their function.

## Objectives and hypothesis

The specific objectives are to compare the experimental group (verbal instructions + body awareness techniques + vaginal palpation) and the control group (verbal instructions + body awareness techniques) with regard to the following factors:

1. The number of fast-twitch muscle fibres, as determined by the number of effective contractions (fast contractions with maximal force lasting 1 s each) out of ten contractions performed [11], before and immediately after the intervention;
2. PFM function (voluntary contraction of the PFMs) and muscular endurance (duration of the muscular contractions in seconds), as measured by the Oxford scale [11], before and immediately after the intervention;
3. The occurrence of associated contractions of the abdominal, gluteal, and adductor muscles during the voluntary contraction of the PFMs before and immediately after the intervention; and
4. Self-efficacy, before and immediately after the intervention, as measured by the self-efficacy scale for the practice of pelvic floor exercises [12].

The hypothesis of the present study is that the experimental group (verbal instructions + body awareness techniques + vaginal palpation) will have significantly more effective fast phasic fibres (contractions assessed by digital palpation) than will the control group after undergoing the interventions, as determined by the primary outcome.

## Methods

### Design

The present study is a single-centre, double-blind (investigators and outcome assessors) randomized controlled trial with two physiotherapy intervention groups. The study was designed following the Standard Protocol Items: Recommendations for Interventional Trials, and the results will be reported in a manner consistent with the consolidated standards of reporting trials (CONSORT) guidelines [13].

### Research site

The research will be conducted in the Urogynaecology and Reconstructive Pelvic Surgery Sector at the Federal University of Sao Paulo in Brazil. The participants will complete one training session per week for 1 month (four sessions) in the urogynaecology clinic. The physiotherapist (PT) will supervise all of the participants during their weekly visits and monitor each subject’s technique, dose (quantity of exercise), and appropriate progression of the exercise programme according to the body and perineal awareness protocol proposed by this study. In addition to the outpatient sessions, the participants will perform the exercises at home.

### Recruitment procedures

Consecutively, included patients with urinary symptoms will be evaluated by a urogynaecologist, who will perform a clinical examination (patient history and physical exam, including a stress cough test) and pad test. Patients with SUI and/or mixed urinary incontinence with predominant SUI symptoms and ≥ 2 g leakage on the pad test will be considered candidates for conservative treatment. The specific type of incontinence will be diagnosed on the basis of clinical parameters, the pad test results [1, 14], and the patients’ reports of which condition bothers them the most (stress urinary or urge incontinence). The patients will then be referred to a PT for the consideration of conservative treatment.

### Participants

The participants will be instructed about the study and asked to provide written informed consent (Additional file 1), which will be obtained by the PT investigator (LAF). The PT (MMG) specialist in rehabilitation of the PFMs will evaluate the patients’ PFM function using vaginal palpation and visually observing the movement of the perineum during contractions. We will include women who have SUI symptoms; have ≥ 2 g of leakage, as measured by a 1-h pad test; have not previously undergone physiotherapy for pelvic floor dysfunction; and have at least grade 1 muscle strength (flicker), as assessed by the two-finger assessment and rated according to the Oxford scale [11]. Patients will not be included if they are younger than 18 years old; have chronic degenerative diseases; have pelvic organ prolapse more severe than stage I, as determined by the POP-Q; have neurologic or psychiatric diseases; have a history of pelvic floor surgeries; or have any intolerance or discomfort in response to the PFM examination.

### Randomization procedures

The participants will be randomly assigned to either the experimental group or the control group. The allocation sequence will be generated by a research assistant (FFF) using a computer-generated random number table with a group ratio of 1:1 and will be concealed in sequentially numbered, sealed, and opaque envelopes. The envelopes will be kept in a closed locker at the centre to which only the research assistant will have access. The envelopes will be given to the PT (LAF) immediately prior to the first outpatient session.

### Blinding

The researchers involved in data acquisition [outcome measures (MMG)], data analysis, and/or statistical analysis (professionals without knowledge of the results) will be blinded to the group allocation. The PT responsible for the physiotherapeutic treatment (LAF) will not be blinded to the allocation of the patients in the groups and, therefore, will not be involved in data acquisition, data analysis, and/or statistical analysis. The participants will not be blinded to the group they are assigned to since the control group will not undergo vaginal palpation as part of the perineal awareness programme. The epidemiological, clinical, allocation, randomization, and outcome data from the study will be stored by the principal investigator in a virtual database to which only he will have access. At the end of the study, the results will be made available to researchers who did not have contact with the participants as well as to statisticians blinded to the groups, and these researchers and statisticians will perform the data analysis.

### Interventions

Both therapy programmes (experimental group, control group) are based on training procedures for motor learning (understanding; searching; learning; control) [7]. The training programme will last for 4 weeks and will include 4 personal physiotherapy consultations. The main difference between the programmes is the applied technique (experimental group: vaginal palpation; control group: verbal instructions). More details on the intervention (therapy plan of the experimental group and control group) are included in Additional file 2. Patients will be encouraged to carry out treatment completely, through text messages (sent weekly by cell phone) that will encourage them to improve their PFM function through their personal effort and will reduce the rate to dropouts. In the event that dropouts of more than 15% are observed, we will perform an intention-to-treat analysis.

There are no known side effects/complications related to the techniques applied, other than possible discomfort following the intervention, and any adverse events will be monitored. All participants will be instructed to contact research personnel if they experience any adverse events at any point during the study.

#### Experimental group: verbal instructions + body awareness techniques + vaginal palpation

For the experimental group, the PT will provide verbal instructions to the patients about the anatomy and function of the PFMs, bladder function, and micturition; instruct them on how to perform body perception training, which includes posture and breathing movements; and instruct them on how to perform PFM contractions by using vaginal palpation as part of the treatment.

The treatment programme is based on motor learning concepts related to the PFMs [7, 15]. The steps of learning the proper muscle contractions will be grouped into the following four phases: (1) the understanding phase, as patients need to understand where the PFMs are located and how they work (cognitive function); (2) the searching phase (“Where is my pelvic floor?”), as patients need time to apply this understanding to their bodies and find where the PFMs are located, though they often need the PT to confirm the location; (3) the learning phase, as patients need to learn how to perform PFM contractions correctly after they find the PFMs, and feedback from the PT is mandatory at this point; and (4) the control phase, as most subjects will still need to work towards performing controlled and coordinated contractions, recruiting as many motor units as possible during each contraction, after having learned to contract the PFMs properly [7]. The awareness programme will last for 4 weeks and will include four outpatient consultations lasting 60 min each (1 session per week).

#### Control group: verbal instructions + body awareness techniques

For the control group, the PT will provide verbal instructions to the patients about the anatomy and function of the PFMs, bladder function, and micturition; instruct them on how to perform body perception training, which includes posture and breathing movements; and instruct them on how to perform PFM contractions without vaginal palpation during the treatment. After the end of the 4-week period, the patients will undergo the same protocol as the experimental group.

After the awareness phase with the protocol including vaginal palpation, the patients in both groups will continue PFMT for SUI management.

### Standardization of treatment

The PT who will be delivering the interventions is extensively trained in administering the standardized treatment protocols and rigorous procedures used for both the experimental and control groups. Routinely, during the course of the study, the PT will meet with the study team to ensure consistency in the protocol and to discuss any concerns that may arise.

### Primary and secondary outcome measures and assessment points

The patients will be evaluated at baseline (*week 0*) and at 4 weeks post-intervention (*week T5*) (Table 1). The study population will be characterized based on sociodemographic [age, body mass index (BMI), duration of symptoms, number of pregnancies, number of vaginal deliveries, hormone therapy (HT), ethnicity, and educational level] and clinical (PFM function and self-perceptions of the effectiveness of the perineal exercises) variables.

**Table 1:**
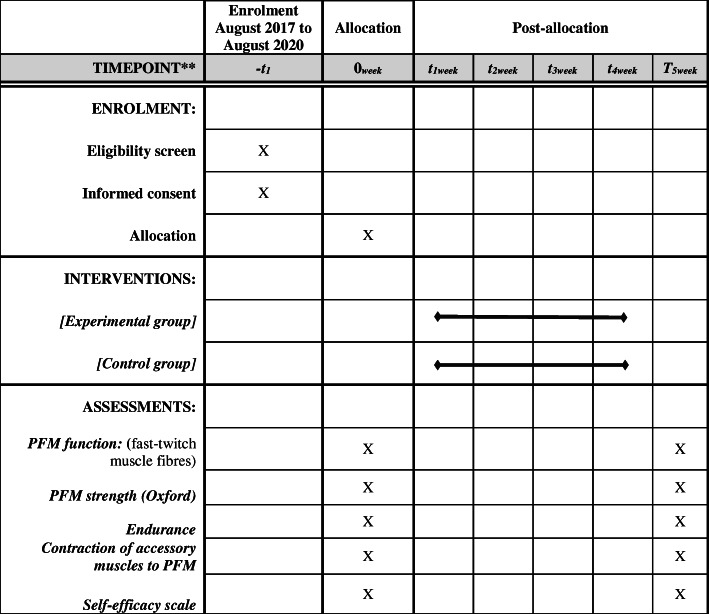
Schedule of enrolment, interventions, and assessments

#### Primary outcome measure

##### Vaginal palpation and visual observation: phasic fibres

The patients will be in the lithotomy position when the phasic fibres of the PFMs are evaluated; they will have their hips and knees semi-flexed and rotated externally and their upper limbs placed to the sides of the trunk. They will be asked to contract their PFMs by raising the pelvic floor musculature towards the pubic symphysis.

The patients will be instructed to perform the contractions quickly and with the maximum possible force 10 times [11]. The number of fast-twitch contractions will be determined by calculating the number of effective contractions (fast-twitch contractions with maximal force lasting 1 s each) out of 10 contractions performed.

#### Secondary outcome measures

The secondary outcome measures will be PFM function and self-efficacy in practising PFM exercises.

##### Vaginal palpation and visual observation

The lithotomy position (with the patient’s hips and knees semi-flexed and rotated externally and their upper limbs placed to the sides of the trunk) will be used to evaluate the contraction and function of the PFMs. The PFM contractions will be assessed using vaginal palpation and visual observation as follows:

1. For the voluntary contractions of the PFMs, the following verbal command will be provided: “squeeze the PFMs as hard as you can”. The scores will be as follows: 1 = no contractions; 2 = weak; 3 = normal; and 4 = strong.
  - PFM function will be assessed using the modified Oxford grading scale [0 = no contractions; 1 = flicker; 2 = weak; 3 = moderate (with lift); 4 = good (with lift); and 5 = strong (with lift)] [11].
  - The occurrence of associated contractions of the abdominal, gluteal, and adductor muscles during the voluntary contraction of the PFMs will also be evaluated.
2. Endurance will be evaluated based on the duration of the muscular contraction in seconds, which reflects the activity of the slow muscle fibres. Ideally, the contractions should be maintained for more than 10 s [11].

##### Self-efficacy scale for practising PFM exercises

The patients’ self-perceptions of the effectiveness of the perineal exercises will be evaluated using the self-efficacy scale for the practice of pelvic floor exercises [12]. The scale is composed of 17 questions provided in a visual analogue scale format, with responses ranging from 0 (not confident) to 100 (most confident). There are 13 questions on self-efficacy and four on the patients’ expectations of the results. The final score is obtained by averaging the scores for the items, and the total score ranges from 0 to 100, where higher values indicate greater self-efficacy/higher expectations regarding the results of training of the PFMs. The 13 questions referring to self-efficacy will be asked after the intervention period only, and the four questions regarding the expectations of the results will be asked before and after the intervention period.

### Trial management

The principal investigator (PI) (LFA) and a research coordinator (FFF) will regularly communicate (through emails, telephone, or in person) to promote and monitor the recruitment progress. The PI, the evaluators, and the physiotherapists will conduct conference calls or face-to-face meetings to monitor the study’s progress. All members of the research team will be informed of the progress through e-mail every 2 weeks. All the data collected will be anonymized and stored in a locked cabinet in Urogynaecology and Reconstructive Pelvic Surgery Sector at the Federal University of Sao Paulo. After each assessment and on the same day, the files will be reviewed by the research assistant to identify missing data. Any missing information will be retrieved immediately by research assistants directly from the study participants. The data will be entered weekly (depending on recruitment rate) into a computerized database, SPSS [Statistical Package for Social Sciences (SPSS Inc., Chicago, IL, EUA)], version 22.0. A final quality-control step will be performed at the time of the data analysis by the trial statistician. Frequency distributions and ranges will be analysed to detect any outliers that could signal potential errors. The data will be analysed without any nominative identifiers.

### Adverse events

In the current study, there are no anticipated risks or inconveniences, as the applied interventions and examinations are well established and widely applied in standard pelvic floor physiotherapy. All of the women will be asked during every physiotherapy and measurement consultation whether they are experiencing any adverse effects. If there are any complications or any complications are suspected, the patients will be instructed to contact a member of the study team, and they will be informed of the procedure to follow for other episodes. The Trial Steering Committee (TSC) is made up of three researchers (MMG, MMPM, and RAC); they will meet weekly and analyse the ongoing results of the research. They will have the power to partially or completely halt the course of the study. No public organizations were involved in the development of this study.

### Statistical analysis and sample size calculation

#### Calculation of the sample size

A sample size calculation was performed to estimate the number of participants needed to obtain a statistical power of 0.80 at an alpha level of 0.05. A pilot study was previously conducted to calculate the sample size (unpublished). The results of this study showed improvements in the fast-twitch muscle fibres in both groups after the intervention. In the experimental group, the mean number of fast-twitch muscle fibres was 1.2 (± 0.7), and in the control group, it was 1.1 (± 1.7). Based on these results, we used Student’s bilateral *t* test to calculate the sample size, which was a minimum of 72 participants per group. To estimate the sample loss during the study, loss percentages from 0 to 20% were assumed. For each patient that is expected to terminate their participation in the study, two new patients will be included. Thus, considering a loss of 20%, the sample size was a minimum of 86 patients in each group.

#### Data analysis

The software SPSS [Statistical Package for Social Sciences (SPSS Inc., Chicago, IL, EUA)], version 22.0, will be used for the statistical analyses. Descriptive statistics will be calculated and tabulated to characterize the participants. For the comparisons of the two means, Student’s *t* tests will be used to compare groups in terms of the quantitative variables that have Gaussian distributions, and the non-parametric Mann-Whitney test will be used to compare groups in terms of the quantitative variables that do not have Gaussian distributions. The qualitative variables will be described as counts and frequencies.

A general linear model (parametric) will be used to compare groups in terms of the quantitative variables that represent the differences between the baseline and post-intervention points, and when it is not possible to use a general linear model, the non-parametric Wilcoxon paired test will be used to compare the quantitative differences between the baseline and post-intervention points in each group separately. The quantitative variables will be described as mean, standard deviation, median, minimum and maximum values, and the number of valid observations (*n*).

The chi-square test (or Fisher’s exact test or the likelihood ratio) will be used to compare the qualitative variables between groups. The generalized estimating equation model will be used to compare groups in terms of the qualitative differences between the baseline and post-intervention time points. When it is not possible to use a generalized estimation model, the non-parametric McNemar test will be used to compare the qualitative differences between the baseline and post-intervention time points in each group separately. The level of significance will be 5% (*p* value < 0.05) for each of the comparisons.

## Results

The data for this trial is currently being collected, and the trial is registered at ClinicalTrials.gov, NCT 03325543. Enrolment is expected to continue through 2019, with long-term outcomes being assessed completely by 2020 for all subjects.

## Discussion

PFMT is the first line of treatment for stress urinary incontinence, as evidenced by high-quality studies. Individuals need to undergo training to contract their PFMs properly, and knowledge and awareness of the PFMs are crucial for effective contractions. However, there is a high prevalence of women who are unable to correctly contract their PFMs. Teaching these women how to properly contract their PFMs is one of the most difficult tasks physiotherapists who work in women’s health and the pelvic floor face, and they often need to use therapeutic methods in their clinical practice to facilitate voluntary and proper PFM contractions [16].

While a proper PFM contraction performed during a vaginal palpation exam feels like a tightening, lifting, and squeezing action under the examining finger, there is no consensus regarding the best method to facilitate PFM contractions. Studies have attempted to determine the most efficacious PFM training programmes, but the results have been inconsistent [16, 17].

This study will determine whether the addition of vaginal palpation into a programme that involves verbal instructions and body awareness techniques can help increase patients’ knowledge and awareness of the pelvic region and increase their ability to perform PFM contractions properly, as required for SUI treatment. Motor re-learning depends on sensory feedback, and learning is, in general, facilitated by the use of feedback. The PT will give external feedback as part of the intervention. The verbal instructions will be based on knowledge of PFM function, and vaginal palpation is a proprioceptive stimulus that can be used to promote learning and help patients properly perform voluntary contractions of PFMs [7, 17].

### Dissemination of study findings

The results of this study will be disseminated through national and international scientific and professional conferences, in addition to undergraduate and postgraduate courses on PFM rehabilitation for physiotherapists.

### Trial status

This study describes version 1 of the protocol (24 March 2020). This trial is actively recruiting participants (165/172). The trial is ongoing and has a planned duration of 3 years, with recruitment continuing from August 2017 to August 2020. If any changes need to be made to the study protocol, the relevant parts of the study will be updated, and the changes will be recorded in the clinical trials registry (ClinicalTrials.gov, NCT 03325543).

## Supplementary information

**Additional file 1:.** Informed consent form.

**Additional file 2:.** Details of the experimental and control groups

## Data Availability

The data analysed during the current study are available from the corresponding author upon reasonable request. The data will be available after the main manuscript is published; for other circumstances, individuals should consult the corresponding author. Any data used to support the protocol can be provided upon request.

## Abbreviations

SUI: Stress urinary incontinence
PFMT: Pelvic floor muscle training
PFM: Pelvic floor muscle
PT: Physiotherapist
PI: Principal investigator
TSC: Trial Steering Committee
