# Supplementary material for: The role of vaginal palpation in motor learning of the pelvic floor muscles for women with stress urinary incontinence: study protocol for a randomized controlled trial
Source: Trials. 2020 Jul 31;21:693. doi: 10.1186/s13063-020-04624-4 (PMC7393708; doi:10.1186/s13063-020-04624-4)
Supplement: Supplementary file 1 — Additional file 1:. Informed consent form. [file 13063_2020_4624_MOESM1_ESM.docx]

**TERM OF INFORMED CONSENT**

We are inviting you to participate in a research project. So that you can decide, it is important to understand what is being studied and what your participation will entail. We will also clarify any questions you may have about the study.

**Title: The role of vaginal palpation in motor learning of the pelvic floor muscles for women with SUI: a study protocol for a RCT**

This information is provided for your voluntary participation in this study, the aim of which is to evaluate the effect of body awareness and awareness of the muscles of the vagina on function of the pelvic floor muscles (muscles of the vagina), quality of life and your perception of the effectiveness of the pelvic floor muscle exercises for the treatment of urinary incontinence.

After the urogynecological checkup with the physician, you will be sent to a physiotherapist, who will conduct an interview as well as assess urine loss and muscle function in the region of the vagina (performed in the gynecological position). You will also answer a questionnaire addressing your confidence with regards to performing the exercises. You will then be sent for treatment involving exercises for the muscles of the vaginal region.

During the interview, information will be collected on your general information (name, age, marital status, profession, schooling) and what problems you are facing due to urine loss. A urine loss evaluation will then be performed with the absorbent test. You will be asked to empty your bladder and then wear an absorbent pad. You will ingest 500 ml of water and then perform exercises (going up and down stairs, jumping, squatting, coughing, walking for 15 minutes and washing your hands for one minute).

This will be followed by an evaluation of the muscles of the vagina in the gynecological position. The evaluation will be performed manually (finger insertion) and you will be asked to contract the muscles of the vagina.

After the evaluations, you will be included in one of the study groups:

- **Experimental group:** This group will receive supervision of the physical therapist that will provide verbal instructions about the anatomy and function of the PFM, bladder function and micturition act, body perception training that includes posture and breathing movements, and how to perform the PFM contractions by using vaginal palpation as part of the treatment.
- **Control group:** This group will receive supervision of the physical therapist that will provide verbal instructions about the anatomy and function of the PFM, bladder function and micturition act, body perception training that includes posture and breathing movements, and how to perform the PFM contractions but with no use of the vaginal palpation tool during the treatment. After the end of the four-week period, the patients will receive the same protocol as the experimental group.

After four weeks of counseling and vaginal muscle training, the final evaluations will be performed (described above). After these evaluations, you will be sent for physiotherapeutic treatment for urine loss.

The evaluations and treatment pose no risks, but there may be discomfort, since you will be evaluated in the gynecological position. A sheet will be used to cover the body prior to the procedure to avoid exposing the patient.

The data obtained with this study will enable demonstrating how counseling, body awareness and awareness of the pelvic floor muscles influence the function of the muscles of the vagina in women with stress urinary incontinence.

Through every step of the study, the researchers will be available to answer any questions and clarify any doubts you may have. The physiotherapist Letícia de Azevedo Ferreira, who can be found at the Urogynecology Clinic located at 1570 Loefgreen Street, Vila Clementino, São Paulo, SP, Brazil, will be available for all clarifications. If you have any questions or doubts about the ethics of the study, contact the Human Research Ethics Committee: 572, Botucatu Street, 572 – 1^st^ floor - 55711062, FAX: 5539-7162, e-mail: cepunifesp@epm.br .

Your participation is voluntary. Therefore, you may withdraw from the study at any time with no negative consequences in terms of your follow up and treatment at the clinic.

Individual information collected during the interview will remain confidential (the women who participate in the study will not be identified.

All participants have the right to be updated regarding the results of the study.

There are no personal expenses for the participants in any phase of the study, including exams and visits with the gynecologist. There will also be no payment for your participation. If any additional expense should arise, it will be paid through the budget allotted to the study.

In cases of personal injury caused directly by the procedures proposed for this study, the participant will have the right to medical treatment as well as any legally established compensation.

The researcher promises to use the data and material collected only for this study.

I have discussed my decision to participate in this study with the physiotherapist Letícia de Azevedo Ferreira. The objectives, procedures, risks and assurances of confidentially and permanent clarifications are clear to me. It is also clear that my participation will not incur expenses and that I have the right to hospital care, if necessary. I voluntarily agree to participate in this study and understand that I may withdraw my consent at any time with no penalties, negative consequences or the loss of any benefit that I may have acquired or the care that I receive at this health service.

_______________________________________________________________

Signature of patient/legal representative Date ____/_____/____

_______________________________________________________________

Signature of witness Date ____/_____/_____

I hereby declare that I have obtained the informed consent of the patient or legal representative in an appropriate and voluntary manner for participation in this study.

_______________________________________________________________

Signature of researcher in charge of study Date ___/_____/_____
