# Supplementary material for: The role of vaginal palpation in motor learning of the pelvic floor muscles for women with stress urinary incontinence: study protocol for a randomized controlled trial
Source: Trials. 2020 Jul 31;21:693. doi: 10.1186/s13063-020-04624-4 (PMC7393708; doi:10.1186/s13063-020-04624-4)
Supplement: Supplementary file 2 — Additional file 2:. Details of the experimental and control groups [file 13063_2020_4624_MOESM2_ESM.docx]

**1^st^ week of exercise program**

1^st^ physiotherapy session

- Goal: Understanding - The patient should understand where the pelvic organs and pelvic floor muscles (PFM) are located and what their individual functions are.

| **Experimental Group** | **Control Group** |
| --- | --- |
| - Information will be provided on:   - PFM anatomy and function, using figures illustrating the PFM and adjacent musculature (gluteal, adductor and abdominal muscles); -Bladder function (bladder filling and emptying phases), showing the patient the behaviour of the PFM in bladder functioning; - Positioning and voiding habits; - Urinary incontinence definition and main risk factors, as well as the impact of a lack of knowledge of the PFM and the risk factors related to the muscle function of the pelvic floor.^1-3^   - Throughout the session, the patient will be able to address any doubts and ask questions about the information provided. | - Information will be provided on:   - PFM anatomy and function, using figures illustrating the PFM and adjacent musculature (gluteal, adductor and abdominal muscles); - Bladder function (bladder filling and emptying phases), showing the patient the behaviour of the PFM in bladder functioning; - Positioning and voiding habits; - Urinary incontinence definition and main risk factors, as well as the impact of a lack of knowledge of the PFM and the risk factors related to the muscle function of the pelvic floor.^1-3^   - Throughout the session, the patient will be able to address any doubts and ask questions about the information provided. |

- Home exercise program:

| **Experimental Group** | **Control Group** |
| --- | --- |
| - Patients will be instructed to start changing their positioning and voiding habits. | - Patients will be instructed to start changing their positioning and voiding habits. |

**2^nd^ week of exercise program**

2^nd^ physiotherapy session

- Goals: Searching - The patient will learn where the PFM are in her body: “Where is my pelvic floor?”

Discovering - The patient will find her PFM.

| **Experimental Group** | **Control Group** |
| --- | --- |
| - Body perception training in supine position with legs flexed, feet supported, back in contact with the table and pelvis in neutral position. - Diaphragmatic breathing with the patient lying supine with legs flexed and feet supported; the patient directs the air towards the ribcage and contracts the transverse abdominal muscle, providing better postural support; - Desensitization of the perineal region (patient in lithotomy position):   - Using the exam spatula, the therapist touches the patient’s perineal region (vulva, tendinous centre of the perineum and external region of the anal canal) and asks the patient to identify the region being touched.  **-** Using digital palpation, the therapist touches the tendinous centre of the perineum, pressing it in the cranial direction, and instructs the patient to contract the PFM.   - The number of contraction repetitions performed will be based on the muscle responses of each patient. - To perform the contraction, the patient is instructed to contract the perineal muscles "by exerting force to contract the vagina, moving it up", so that she understands the force that must be exerted.^2^ - The actions of the abdominal, adductor and gluteal muscles should be suppressed per verbal instructions.^4^ - Throughout the session, the patient will be able to clarify any doubts and ask questions about the information provided. | - Body perception training in supine position with legs flexed, feet supported, back in contact with the table and pelvis in neutral position. - Diaphragmatic breathing with the patient lying supine with legs flexed and feet supported; the patient directs the air towards the ribcage and contracts the transverse abdominal muscle, providing better postural support; - PFM contraction guidance:   - The patient stays dressed and lies supine with the legs flexed and feet supported; the patient is verbally instructed to contract the perineal muscles "by exerting force to contract the vagina, moving it up", so that she understands the force that must be exerted.^2^   - The actions of the abdominal, adductor and gluteal muscles should be suppressed per verbal instructions.^4^ - Throughout the session, the patient will be able to clarify any doubts and ask questions about the information provided. |

- Home exercise program:

| **Experimental Group** | **Control Group** |
| --- | --- |
| Aim: motor learning  Exercises: isolated PFM contractions  Muscle action: concentric, slow to moderate  Repetitions: 10  Sets: 3  Rest: -  Frequency: 3x/day - every day  Position: supine | Aim: motor learning  Exercises: isolated PFM contractions  Muscle action: concentric, slow to moderate  Repetitions: 10  Sets: 3  Rest: -  Frequency: 3x/day - every day  Position: supine |

**3^rd^ week of exercise program**

3^rd^ physiotherapy session

- Goal: Learning - After finding the PFM, the patient will learn to contract them properly.

| **Experimental Group** | **Control Group** |
| --- | --- |
| - PFM contraction training:   **-** Myotatic stretch reflex: With the patient in the lithotomy position, the physiotherapist uses two-finger palpation (with the index and middle fingers inserted 3 cm distally) in the vaginal canal and performs rapid downward stretching of the levator ani muscles, internally, on the posterior wall of the anus. First, this is performed without contraction of the PFM. Then, the patient is asked to contract the muscles associated with the myotatic stretch reflex.^2, 4, 5^   - Body perception training while seated on a rigid surface with bone contacts (ischial tuberosity) and the completion of pelvic movements (anteversion, retroversion and rotations).   - Without two-finger palpation and while sitting on a rigid surface, the patient is instructed to contract the PFM. This contraction exercise is repeated 10 times, with a rest time of 1-2 minutes between contractions.   - Throughout the session, the patient will be able to clarify any doubts and ask questions about the information provided. | - Body perception training while seated on a rigid surface with bone contacts (ischial tuberosity) and the completion of pelvic movements (anteversion, retroversion and rotations). - PFM contraction training (with the patient dressed and seated on a rigid surface):   - Without two-finger palpation and while sitting on a rigid surface, the patient is instructed to contract the PFM. This contraction exercise is repeated 10 times, with a rest time of 1-2 minutes between contractions.   - Throughout the session, the patient will be able to clarify any doubts and ask questions about the information provided. |

- Home exercise program:

| **Experimental Group** | **Control Group** |
| --- | --- |
| Aim: motor learning  Exercises: isolated PFM contractions  Muscle action: concentric, slow to moderate  Repetitions: 10  Sets: 3  Rest: -  Frequency: 3x/day - everyday  Position: supine and seated | Aim: motor learning  Exercises: isolated PFM contractions  Muscle action: concentric, slow to moderate  Repetitions: 10  Sets: 3  Rest: -  Frequency: 3x/day - everyday  Position: supine and seated |

**4^th^ week of exercise program**

4^th^ physiotherapy session

- Goal: Control - After learning how to correctly contract the PFM, the patient should be­­ able to control, coordinate and perform the contractions in different positions.

| **Experimental Group** | **Control Group** |
| --- | --- |
| - First, the patient stays in the lithotomy position to determine if she is contracting the PFMs correctly; adjustments should be performed if necessary. - Body perception training in a standing position with the feet supported, back against the wall and pelvis in a neutral position. - PFM contraction training (supine, seated and standing):   - Supine: with the back supported, legs flexed and arms alongside the body, the patient contracts the PFM. This contraction exercise is repeated 10 times, with a rest time of 1-2 minutes between contractions.  - Seated: on a rigid surface (with the legs apart, feet on the ground, hips flexed and back straight), while keeping the perineal region in contact with the surface, the patient contracts the PFM. This contraction exercise is repeated 10 times, with a rest time of 1-2 minutes between contractions.  - Standing: with the back against the wall, legs semi-flexed and not supported by the wall, while keeping the posture correct, the patient contracts the PFM. This contraction exercise is repeated 10 times, with a rest time of 1-2 minutes between contractions.^4, 6^   - Throughout the session, the patient will be able to clarify any doubts and ask questions about the information provided. | - Body perception training in a standing position with the feet supported, back against the wall and pelvis in a neutral position. - PFM contraction training (supine, seated and standing):   - Supine: with the back supported, legs flexed and arms alongside the body, the patient contracts the PFM. This contraction exercise is repeated 10 times, with a rest time of 1-2 minutes between contractions.  - Seated: on a rigid surface (with the legs apart, feet on the ground, hips flexed and back straight), while keeping the perineal region in contact with the surface, the patient contracts the PFM. This contraction exercise is repeated 10 times, with a rest time of 1-2 minutes between contractions.  - Standing: with the back against the wall, legs semi-flexed and not supported by the wall, while keeping the posture correct, the patient contracts the PFM. This contraction exercise is repeated 10 times, with a rest time of 1-2 minutes between contractions.^4, 6^   - Throughout the session, the patient will be able to clarify any doubts and ask questions about the information provided. |

- Home exercise program:

| **Experimental Group** | **Control Group** |
| --- | --- |
| Aim: motor learning  Exercises: isolated PFM contractions  Muscle action: concentric, slow to moderate  Repetitions: 10  Sets: 3  Rest: -  Frequency: 3x/day - everyday  Position: supine, seated and standing | Aim: motor learning  Exercises: isolated PFM contractions  Muscle action: concentric, slow to moderate  Repetitions: 10  Sets: 3  Rest: -  Frequency: 3x/day - everyday  Position: supine, seated and standing |

**References**

1. Kasawara KT, Oliveira JM, Carneiro MCAS, Dias LB, Junior NC. Assessing knowledge on pelvic floor muscles and role of physiotherapy in urogynecology among Internet users. HealthMED. 2015;9(10):419-424.
2. Bø K, Mørkved S. Pelvic floor and exercise science. In: Bø K, Bary B, Siv M, Marijke VK, editors. Evidence-based Physical Therapy for the Pelvic Floor: Bridging science and clinical practice. 2 ed. Toronto: Elsevier; 2015. p. 111-115.
3. Brown C. Pelvic floor reeducation: a pratical approuch. In: Corcos J, Schick E. The Urinary Sphincter. Marcel Dekker: Nova York; 2013. p. 459-474.
4. Chiarapa TR, Cacho DP, Alves AFD. Tratamento Fisioterápico. In: Chiarapa TR, Cacho DP, editores. Incontinência Urinária Feminina: assistência fisioterapêutica e multidisciplinar. São Paulo: Livraria Médica Paulista; 2007. p. 133-146.
5. Bø K, Finckenhagen HF. Vaginal palpation of pelvic floor muscle strenght: inter-test reproducibility and comparison between palpation and vaginal squeeze pressure. Acta Obstet Gynecol Scand. 2001;80(10):883-887.
6. Sapsford RR, Richardson CA, Maher CF, Hodges PW. Pelvic floor muscle activity in different sitting postures in continent and incontinent women. Arch Phys Med Rehabil. 2008;28:1741-1747.
